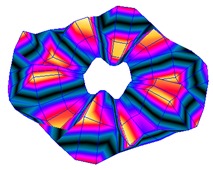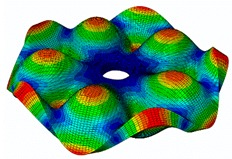# Experimental-Numerical Design and Evaluation of a Vibration Bioreactor Using Piezoelectric Patches

**DOI:** 10.3390/s19020436

**Published:** 2019-01-21

**Authors:** David Valentín, Charline Roehr, Alexandre Presas, Christian Heiss, Eduard Egusquiza, Wolfram A. Bosbach

**Affiliations:** 1 Center for Industrial Diagnostics and Fluid Dynamics (CDIF), Polytechnic University of Catalonia (UPC), 08034 Barcelona, Spain; alexpresas@hotmail.com (A.P.); eduard.egusquiza@upc.edu (E.E.); 2 Experimental Trauma Surgery, Justus Liebig University of Giessen, 35390 Giessen, Germany; Charline.Roehr@med.uni-giessen.de (C.R.); christian.heiss@chiru.med.uni-giessen.de (C.H.); wolfram.bosbach@med.uni-giessen.de (W.A.B.); 3 Department of Energy and Power Engineering, Tsinghua University, Beijing 100084, China

**Keywords:** bioreactor, vibration, cell biology, PZTp, laser Doppler vibrometer (LDV), ultrasound, finite element method

## Abstract

In this present study, we propose a method for exposing biological cells to mechanical vibration. The motive for our research was to design a bioreactor prototype in which in-depth in vitro studies about the influence of vibration on cells and their metabolism can be performed. The therapy of cancer or antibacterial measures are applications of interest. In addition, questions about the reaction of neurons to vibration are still largely unanswered. In our methodology, we used a piezoelectric patch (PZTp) for inducing mechanical vibration to the structure. To control the vibration amplitude, the structure could be excited at different frequency ranges, including resonance and non-resonance conditions. Experimental results show the vibration amplitudes expected for every frequency range tested, as well as the vibration pattern of those excitations. These are essential parameters to quantify the effect of vibration on cell behavior. Furthermore, a numerical model was validated with the experimental results presenting accurate results for the prediction of those parameters. With the calibrated numerical model, we will study in greater depth the effects of different vibration patterns for the abovementioned cell types.

## 1. Introduction

In our present study, we work on the design of a bioreactor in which we want to expose cell lines to mechanical vibration [1]. This research is of great importance as it contributes to the understanding of phenomena in cell biology that might be usable in the future for novel medical therapies. High-intensity focused ultrasound is already today used in tests for the treatment of cancer [2,3,4,5]. The vibration behavior of bacteria can be used in devices for their detection, which is of great importance with regards to sterile hospital conditions [6,7,8]. More importantly, antibacterial measures by ultrasound are also possible where the resonance of cells decreases proliferation rates [9,10]. A very simple but highly interesting experiment was published in Reference [11]. It shows the change of resonance behavior in *Escherichia coli* bacteria after genetic modifications. The processes on a cellular level involved in the forming of responses are still largely unknown. It is known that also DNA absorbs vibration and can be altered by this [12]. In the clinical context, the application of vibration to osteosarcoma cells is one of the leading fields. They were tested in the past for their reaction to ultrasound exposure, particularly in the megahertz range [13,14,15,16]. Research results were published on aspects about patient benefits such as limb salvaging and noninvasive therapy [17,18,19,20]. The beneficial effects of cyclical loadings at low frequencies on the growth of bone tissue [21,22,23,24] and on endothelial cells [25,26] are well documented. These effects are known to be achieved in the hertz range. Vibration in the range of kilohertz influences neurons and can trigger distinct responses from them [27,28]. With our design for a vibration apparatus, in-depth in vitro analyses will be possible. The future results are intended to further the understanding about vibration’s influence on a cellular level.

Piezoelectric patches (PZTp) were widely used in the past [29] in order to excite structures, and also used as energy harvesters [30,31]. Due to the piezoelectric effect [32], PZTps are deformed when a voltage is applied to them. By applying a controlled voltage and attaching the PZTp to a structure, this structure can be excited as desired. If the dynamic response of the structure is known (i.e., its natural frequencies, damping, and mode-shapes [33]), the amplitude and the shape of the vibration when exciting the structure are totally controlled. When a structure is exited in one of its natural frequencies, the amplitude of the vibration is amplified substantially. This phenomenon is known as resonance [33,34]. Many failures in structures were documented along the years due to a resonance phenomenon [35,36].

In this study, a new method of inducing vibration to biological cells is presented. This method is based on the resonance phenomenon of structures. A PZTp attached to a structure is used to determine its dynamic response and, therefore, to know its natural frequencies, damping ratios, and mode-shapes. PZTps are able to induce the resonance phenomenon. Out-of-resonance excitations are equally achievable by PZTps at frequencies far away from natural frequencies. In that way, the amplitude of vibration of the structure is controllable with minimum energy consumption of the PZTp. The structure selected for this study was a bronze disc due to its well-known dynamic response [33,37,38,39,40], its cost, and its possibilities with regards to vibration reactor prototyping.

The dynamic response of discs was studied for many years [41,42,43]. The natural frequencies of discs depend on the material properties and the geometrical characteristics. Each natural frequency has its own damping ratio and is associated to a mode-shape. The mode-shapes are formed by nodal diameters (ND) and nodal circles (NC) [41] which are points that remain stationary during the vibration cycle. Both increase in number with greater frequency. The vibration amplitude for each natural frequency depends on the damping ratio associated with it. The damping ratio of each natural frequency has to be determined experimentally. Numerical models neglect it or only consider it as an unknown input value [33]. Therefore, experiments are always necessary when estimating the vibration amplitude of a structure under resonance conditions and they are essential to calibrate and validate numerical models.

In our present paper, we perform an experimental test for the accurate determination of the dynamic response of a disc in different ranges of frequency. We select three different ranges of frequencies for our investigations, thereby determining, to a great extent, the behavior of cells under vibration: 0–22 kHz, 22–50 kHz, and 50–100 kHz. These ranges were selected in order to quantify the effect of low-, medium-, and high-frequency vibrations on cell behavior. We calibrated a numerical model for the experimental damping and used it for predictions about the dynamic response of the disc. This study is the proof of concept for the design of a vibration bioreactor to study the behavior of different cell lines under vibration such as osteosarcoma cells (OS), bacteria, or neurons.

## 2. Methodology

**Figure 1:**
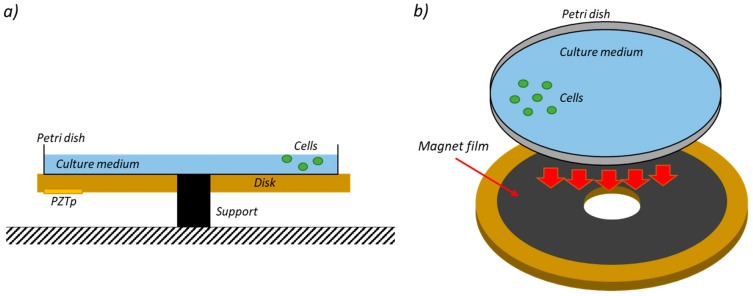
Schematic of the planned bioreactor. (**a**) Cross-section view; (**b**) Assembly of the petri dish.

The sketch of the planned vibrating bioreactor is shown in Figure 1. As can be seen in this figure, the bioreactor is based on a disc with a petri dish over the disk surface. The culture medium with the cells is inside the petri dish, which is installed by means of a magnet film. This allows the petri dish to be removed easily from the disc for examining the cells after each vibration cycle. 

**Figure 2:**
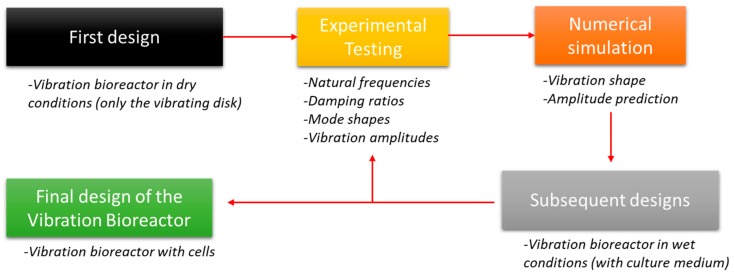
Schematic diagram of the prototyping methodology.

The methodology to develop the proof of concept of the vibration bioreactor is shown in Figure 2. An initial design of the bioreactor in dry conditions was experimentally tested. The experimental set-up was based on a disc instrumented with a PZTp. The disc was excited with this PZTp and its vibrational response was measured with a laser Doppler vibrometer (LDV), as well as with an ultrasound sensor. With this experiment, the natural frequencies, mode-shapes, and damping of the disc could be obtained, as well as the vibration amplitudes in different frequency ranges. After that, a numerical model was built. The numerical model simulated the same conditions as in the experimental test. The numerical model can be calibrated and validated according to the experiment for predicting the vibration amplitudes and vibration shapes of the disc. The bioreactor will also be tested in wet conditions with the petri dish containing the culture medium. Results obtained at this stage will be used to achieve the final design of the vibration bioreactor.

### 2.1. Experimental Investigation

#### 2.1.1. Experiment Set-Up

**Figure 3:**
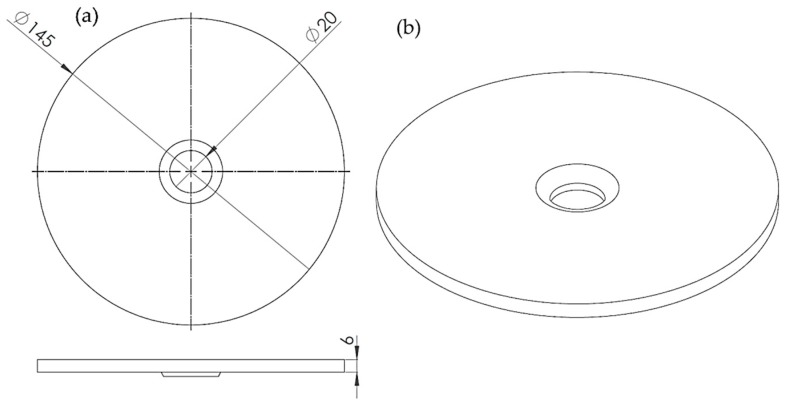
Disc sketch. (**a**) Main dimensions of the disc; (**b**) isometric view of the disc.

**Figure 4:**
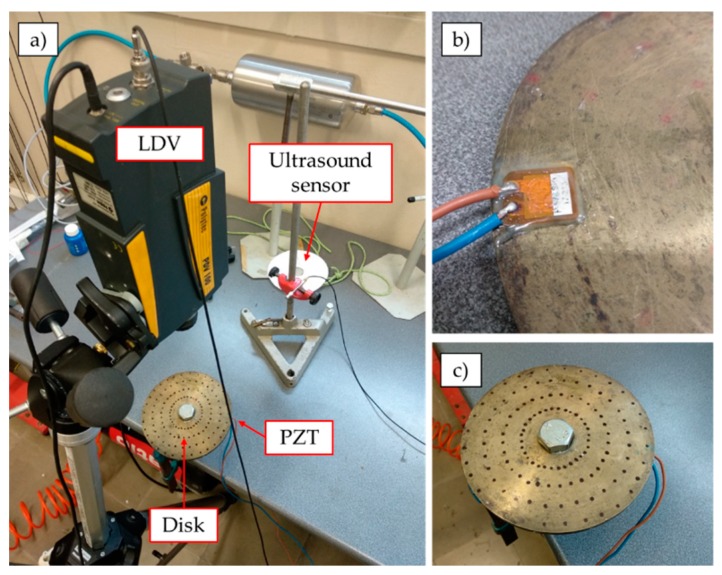
(**a**) Picture of the experimental set-up; (**b**) detail of the piezoelectric patch (PZTp); (**c**) detail of the disc.

The experiment consisted of exciting a disc with one PZTp and measuring its response with an LDV and an ultrasound sensor. In this case, the petri dish was not included. The disc was made of bronze. Its external and internal diameters were 145 mm and 20 mm, respectively. The thickness of the disc was 6 mm (see Figure 3). The disc was supported on its internal radius, so that it could be rotated without any mechanical constraint. Pictures of the experimental set-up are shown in Figure 4.

#### 2.1.2. Instrumentation

The disc was excited by means of the PZTp. This PZTp was a P-876 DuraAct glued to the disc surface with an epoxy component LOCTITE454. The piezoelectric material of the PZTp was PIC255 and it was composed of multiple laminated ceramic layers. It was confirmed that the PZTp’s weight was negligible with regard to the set-up’s vibration response. The PZTp worked in a range of 100–250 V. 

For the signal generation, an NI-9263 module was used. This module can generate four independent analog outputs with an amplitude of −10 to 10 V. With an amplifier OEM-835, the analog signal was amplified by a factor of 25. A signal for monitoring the excitation was sent to the data acquisition system.

To measure the response of the disc, an LDV PDV-100 with adjustable sensitivity (sensitivity range of 200–8 V/(m/s)) was used. The laser was mounted on a tripod without contact to the disc support. The LDV measuring frequency range was 0–22 kHz. Therefore, an ultrasound sensor (PCB 378C01) was used above 22 kHz. This ultrasound sensor can measure up to 100 kHz with a sensitivity of 2 mV/Pa. Both the LDV and the ultrasound sensor were always maintained in the same position.

The three signals (PZTp, LDV, and ultrasound) were acquired simultaneously by an acquisition system Bruel&Kjaer Type 3038, which has multiple channels of an acquisition frequency 65.536 kHz, and one channel of 262.144 kHz. The ultrasound sensor was always connected to the channel with greater acquisition frequency.

#### 2.1.3. Experimental Procedure

A chirp signal was sent to the PZTp to excite the disc [33]. The signal had a duration of 10 s. Three different ranges of excitation were applied: 0 to 22 kHz, 22 to 50 kHz, and 50 to 100 kHz. In that way, the influence of the frequency on cell behavior could be studied in detail.

For the first frequency range in which the LDV could measure the disc response, an experimental modal analysis (EMA) [44] was performed. For this purpose, the disc was marked with different spots (see Figure 4) where the LDV was pointed at. A chirp was sent to the PZTp and the vibrational response of the disc was measured with the LDV at all points (32 points in four different radii of the disc, total of 128 points). To carry out this procedure, the LDV was maintained in the same position for all four radii of points and the disc was manually turned 11.25 degrees every time. 

A frequency response function (FRF) [33] between the LDV signal and the excitation voltage signal of the PZTp was computed for each of the 128 points. The chosen frequency resolution was 0.0625 Hz (period of 16 s) with a maximum frequency analysis of 25.6 kHz. In that way, the mode-shapes of the disk could be accurately determined, which is mandatory for the evaluation of the vibration response. This result is also essential for understanding how cells will vibrate in the bioreactor.

The remaining frequency ranges were beyond the LDV range. Therefore, only the ultrasound sensor measured the vibration response of the disc due to the transmission of the vibration through air. For these cases, the FRF was computed between the ultrasound sensor and the PZTp; however, it was limited to only one position on the disc. The frequency resolution chosen was the same as before (0.0625 Hz). The maximum analysis frequency was 102.4 kHz.

**Figure 5:**
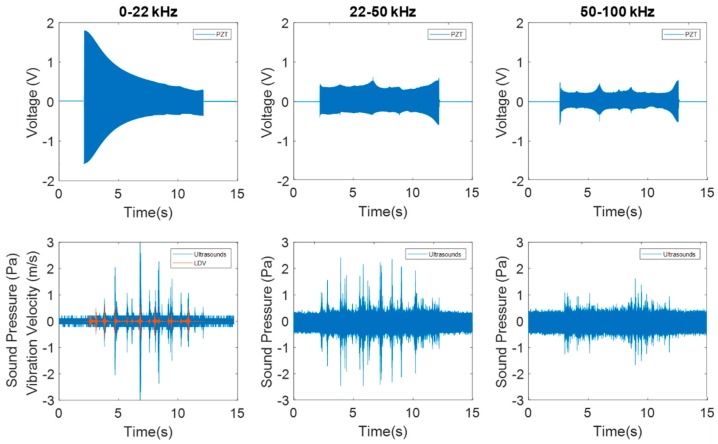
Time signal of the PZTp excitation and the responses of the laser Doppler vibrometer (LDV) and ultrasound sensor for the three different ranges of excitation tested.

Figure 5 shows an example of the experimental procedure. The chirp excitation with the PZTp is shown in volts, and the responses of the disc measured by means of the LDV and the ultrasound sensor are also shown for each frequency range tested.

### 2.2. Numerical Simulation

#### 2.2.1. Finite Element Method (FEM) Model

The numerical model was based on the finite element method (FEM) code of the commercial software package Abaqus® [45]. The disc was meshed with standard and linear hexahedral elements and fixed in its perpendicular direction by constraints to the nodes located on the inside diameter. The density of the disc was simulated according to measurements carried out experimentally with the real disc (ρ_disc_ = 7666 kg/m^3^) and for a Young’s modulus according to bronze’s standard characteristics (E = 10^11^ Pa). The next section describes the simulations performed with this model.

#### 2.2.2. Numerical Procedure

**Figure 6:**
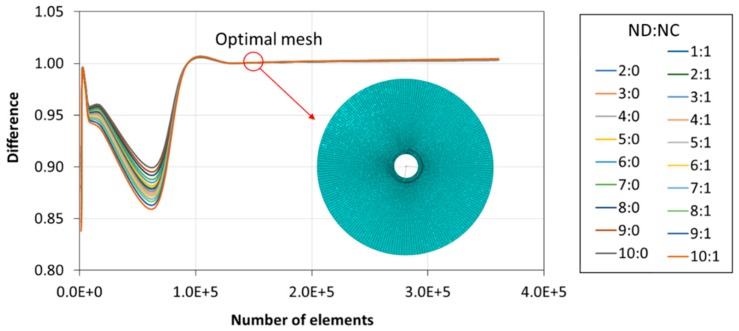
Mesh sensitivity study and optimal mesh selected. Difference obtained from the relationship between the natural frequency in each simulation and the natural frequency with the optimal mesh.

Firstly, a sensitivity analysis of the mesh was carried out. The sensitivity analysis ensured that the results obtained were independent of the element size. For this purpose, the size of the elements was decreased until numerical results obtained in Abaqus were independent of the mesh’s coarseness. Natural frequencies were compared between the set of FEM meshes (for frequencies less than 100 kHz). The mesh selected had 1.3 × 10^5^ elements with an element size of 0.8 mm. The mesh sensitivity analysis and the optimal mesh are shown in Figure 6.

Based on the selected mesh, a harmonic analysis in the desired frequency range was performed. A force of 100 N was applied to the structure in the position where the PZTp was installed for experimental measurements. This value was applied according to the PZTp manufacturer’s characteristics. The vibrational response of the disc was obtained. The damping ratio for each mode-shape obtained by means of the experiment was introduced in the simulation in order to accurately obtain the vibration amplitude.

## 3. Results and Discussion

### 3.1. Experimental Results

#### 3.1.1. Experimental Modal Analysis

**Figure 7:**
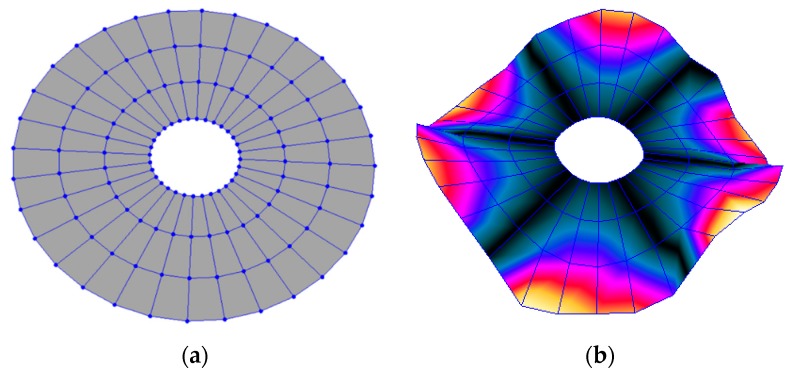
(**a**) Geometry for the operational deflection shape (ODS); (**b**) example of a mode-shape of the disc obtained using the ODS.

The FRF between the LDV and the PZTp was computed for the 128 points measured (see Section 2.1.3 for further details). Each FRF was assigned to a point in a virtual geometry for applying an operational deflection shape (ODS) using the software PULSE Reflex® [46]. Figure 7 shows the virtual geometry used and one mode-shape obtained by means of the ODS.

**Figure 8:**
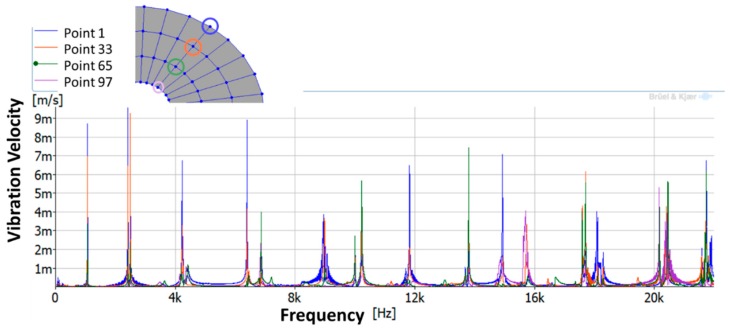
Autospectra of four points located in different radii of the disc during the experimental modal analysis (EMA).

In the analyzed range from 0 to 22 kHz, there were multiple natural frequencies of the disc. Figure 8 shows the fast Fourier transform (FFT) of four different points in different radii of the disc. Multiple peaks can be observed within this range. It can clearly be seen that the difference in amplitude between in-resonance conditions (a natural frequency peak) and out-of-resonance conditions was substantial. Making good use of this variation was key to the bioreactor prototype design. The ODS representation permitted the experimental association of each natural frequency with one mode-shape.

The mode-shapes of the disk were classified according to the numbers of NDs and NCs [38,41]. In that way, each mode-shape could be named as a combination (ND, NC). The values of the natural frequencies and damping ratios associated with each mode-shape found in the range of 0–22 kHz are shown in Table 1. It should be noted that, for modes where a doublet was detected (same mode-shape but with phase difference of 90 degrees and nearby frequency value), only one of the two mode-shapes is contained in the table. The “3db method” [47] was used to calculate the damping ratio from each peak. Results obtained were in agreement with results presented in previous studies about modal analysis in circular diskc [33,34,38].

**Figure 9:**
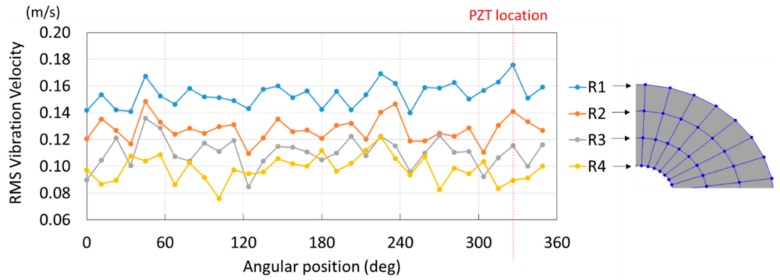
Root-mean-square (RMS) vibration velocity within the range 0–22 kHz for each point of the disc. R1 to R4, from external radius to internal radius of the disk.

As shown in Table 1, the first 10 nodal diameters without nodal circles were found within the frequency range of 0–22 kHz. Only five nodal diameters with one nodal circle were found within this range. This means that the maximum deformation of the disc was located close to the edge of the disc. This finding was of great importance for the bioreactor prototype design. To check that this was true, the energy of vibration at each point could be calculated as the root-mean-square (RMS) value within the studied frequency range. Figure 9 shows the RMS vibration velocity in the range 0–22 kHz of all points measured on the disc and classified by their angular position. It can clearly be seen that the energy was concentrated in the exterior radius of the disc (R1 in Figure 9). Therefore, the optimal position for the cell’s location to obtain maximum vibration energy will be at the external edge and near the PZTp location.

#### 3.1.2. Dynamic Behavior at Higher Frequency Ranges 

The LDV was not able to measure the disc vibration above 22 kHz since it was out of its measuring range. This is why an ultrasound sensor was also used in this experiment. Zhu et al. [48] demonstrated that there is a direct relationship of the vibration of the structure and the acoustic pressure measured by a microphone, or, in this case, an ultrasound sensor. Measuring the high frequency ranges of excitation with the ultrasound sensor gave an idea of the vibration of the disc at those frequencies.

**Figure 10:**
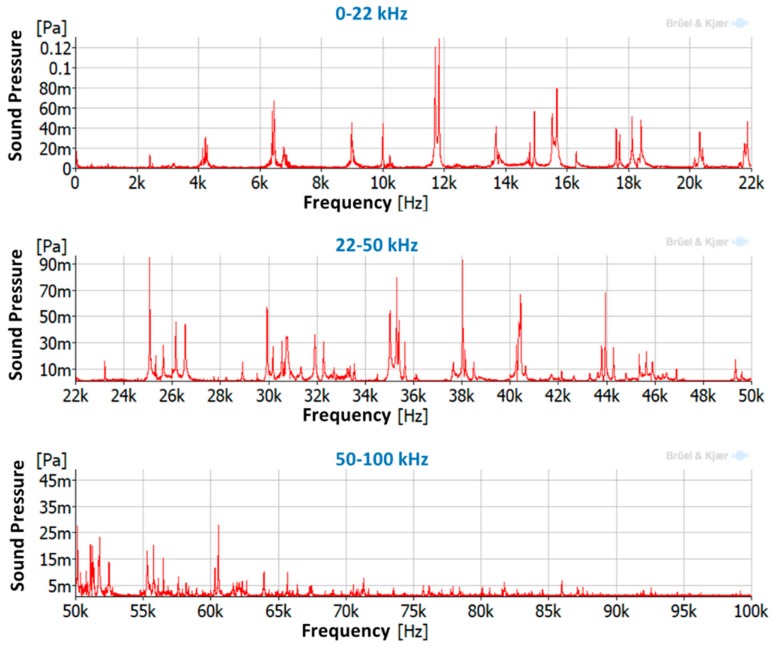
Ultrasound sensor autospectra for different frequency ranges.

Figure 10 shows the autospectra of the ultrasound sensor for the three different ranges of excitation tested. It can be seen that the natural frequencies of the disc were clearly identified in the first range (0–22 kHz) and that they were identical to those found with the LDV. In the second range (20–50 kHz), the natural frequencies of the disc were also clearly identified and with similar amplitude as in the first range, which means that the vibration energy in both ranges was similar. However, the third range of frequency analyzed (50–100 kHz) presented lower amplitudes and the peaks were more difficult to identify. As Watson et al. [49] showed in their work, the vibrational response of structures decreases greatly for high frequencies, in particular from 50 to 300 kHz. This behavior is similar to the results of the studied disc.

With greater natural frequency values, the number of NDs and NCs in the disc mode-shapes increased. As the frequency increased, the deformation in the disc was rather located in single points than on the disc surface. By variation of the excited natural frequency, the influence of the natural frequencies could also be studied. In comparison to previous work where non-contact methods were used to excite the cells, here, the energy exposure of the cells was greater and could be quantified by measuring the vibro-acoustic response of the disc. With the non-contact methods, the actual force applied to the structure is rather small, of about 1 N or less for the high-intensity focused ultrasound therapeutic applications [17,18,19,20]. By means of the proposed method, the PZTp could provide about 100 N according to the manufacturer’s specifications. In addition, exciting the structure in resonance conditions would amplify the vibration amplitude considerably.

### 3.2. Numerical Results

#### 3.2.1. Modal Analysis

The results for the numerical modal analysis are found in Table 2. In this table, both natural frequency values and the mode-shapes are compared with the experimental results. A good agreement was found with an error below 5% between the experiment and the numerical simulation. Only the mode-shapes within the frequency range of 0–22 kHz are shown in this table; however, the analysis was performed up to 100 kHz. Local characteristics of the vibration increased for greater frequency. For the high-frequency mode-shapes, the disc vibrated with maximum amplitude in small areas, which means that the cells should be located in these points of maximum displacement.

#### 3.2.2. Harmonic Analysis

**Figure 11:**
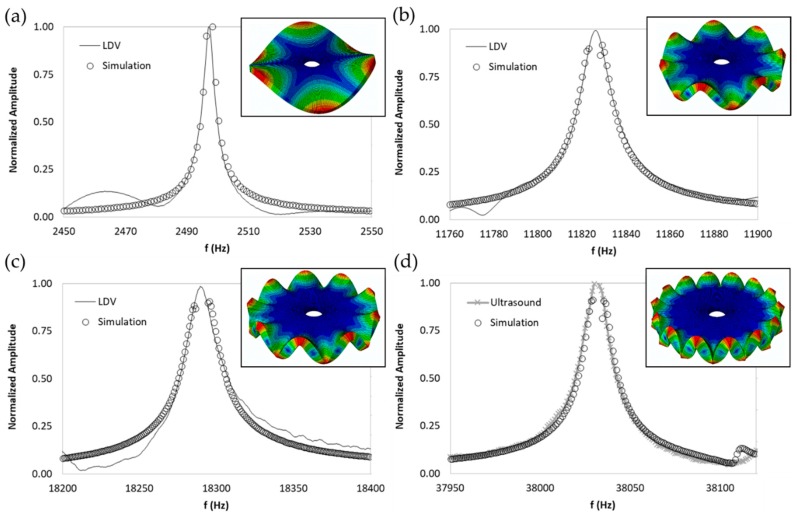
Normalized frequency response function (FRF) of four different mode-shapes. Numerical and experimental comparison. (**a**) (3,0); (**b**) (7,0); (**c**) (9,0); (**d**) (14,0).

The numerical harmonic analysis was performed around the natural frequencies found by means of the modal analysis. The damping ratio obtained with the experiments (see Table 1) was introduced in the numerical model for each mode-shape. Figure 11 shows an example of the results obtained for four different natural frequencies. In the plots (a), (b), and (c), the numerical results are compared with the experimental results obtained with the LDV. In plot (d), the numerical results are compared with the ultrasound sensor since the LDV could not measure at this frequency. The FRF was normalized with the maximum of each peak and the frequency of the simulation was corrected with the error factor shown in Table 2. A good agreement for the amplitude was found between the numerical and the experimental results at the natural frequency, which is the point where the disc will be excited to expose cells to vibration. Therefore, the numerical model was validated and will be useful for further design steps, as well as for the prediction of the vibration amplitude at the natural frequencies on the whole disc surface.

## 4. Conclusions

A vibrating bioreactor apparatus was designed under the concept of resonance vibration. For this, we built a vibrating apparatus based on a bronze circular disc. The disc was instrumented with a PZTp, which was able to excite the structure at different frequencies and amplitudes. Upon exciting the disc in its natural frequencies, the vibration amplitude was amplified substantially which can be used to expose different kinds of cell lines to vibration. In comparison with the current non-contact methods used to expose cells to vibration, the proposed method imposes frequencies from low to high frequency ranges, as well as amplitudes at far greater controllability and reproducibility.

The natural frequencies of the disc were studied in detail by means of experimental and numerical modal analysis. For the experiment, the disc was instrumented with a PZTp, and its vibration response was measured with an LDV and an ultrasound sensor. For the numerical investigation, an FE model of the disk was constructed introducing the experimental values of the damping ratio to calibrate the vibration amplitude. Both numerical and experimental results for natural frequencies and mode-shapes presented good agreement. It was seen that the maximum deformation was located near the tip of the disc. Additionally, with greater natural frequencies, the excitation became more localized. These results are very useful for understanding how the disc vibrates at the different frequencies and, therefore, how the attached cells will vibrate. 

For the disc studied, the vibration amplitude decreased considerably from 50 kHz onward. For these high frequencies, it was demonstrated that the structural response was low; however, it still existed. With the validated numerical model, the disc dimensions can be adapted for the desired frequency ranges to study.

Previous studies with cells exposed to vibration considered high frequency due to ultrasound excitations [13,14,15,16]. In those studies, the force applied to the structure was very low in comparison with that applied with the technique presented in this paper. Furthermore, with the current technique the way how the cells vibrate is highly controllable, whereas, with non-contact methods such as ultrasound, it is rather random. This method also permits studying the low frequency range, which, for some cells such as neurons, will be important.

## Figures and Tables

**Table 1:** Natural frequencies and damping ratios associated to each experimentally obtained mode-shape. ND—nodal diameter; NC—nodal circle.

Natural Frequency (Hz)	Damping Ratio (%)	Mode-Shape	
		ND	NC
1060	0.1520	2	0
2416	0.0660	3	0
4197	0.2240	4	0
6419	0.1610	5	0
8982	0.1050	6	0
11826	0.0540	7	0
14929	0.0440	8	0
18111	0.0530	9	0
21586	0.0470	10	0
6850	0.1370	2	1
10236	0.0710	3	1
13685	0.0490	4	1
17594	0.0230	5	1

**Table 2:** Comparison of experimental modal analysis (EMA) and numerical modal analysis. $Diff\left(\%\right)=100\frac{f_{n,sim}-f_{n,exp}}{f_{n,exp}}$.

Exp (Hz)	Sim (Hz)	Diff %	Mode-Shape			
			ND	NC	Experiment	Simulation
1060	1025	−3.30	2	0	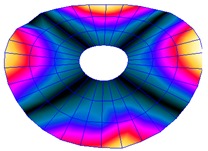	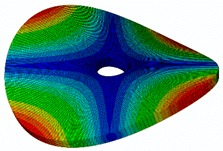
2416	2396	−0.83	3	0	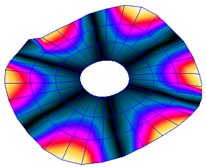	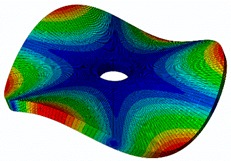
4197	4156	−0.98	4	0	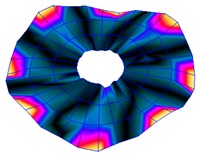	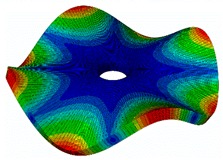
6419	6286	−2.07	5	0	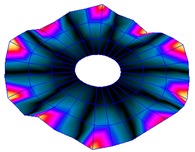	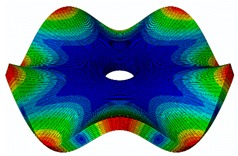
8982	8753	−2.55	6	0	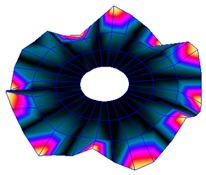	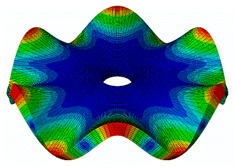
11826	11525	−2.55	7	0	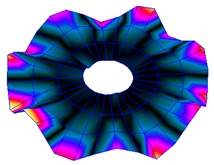	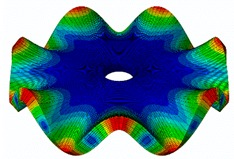
14929	14570	−2.40	8	0	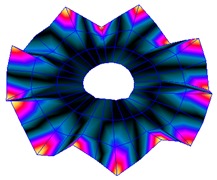	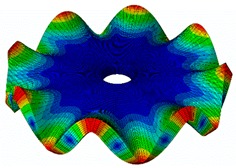
18111	17859	−1.39	9	0	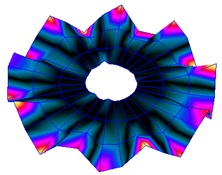	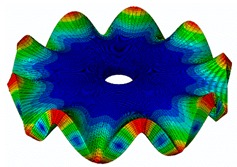
21586	21365	−1.02	10	0	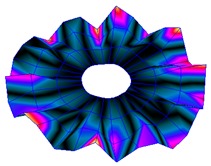	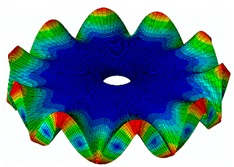
6850	6561	−4.22	2	1	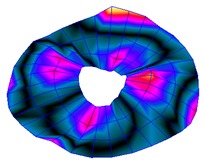	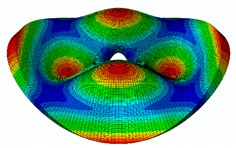
10236	9764	−4.61	3	1	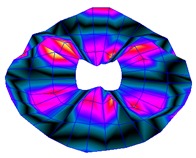	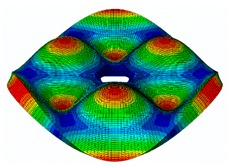
13685	13267	−3.05	4	1	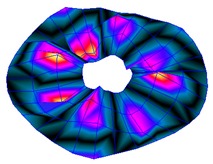	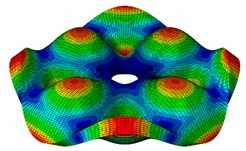
17594	17056	−3.06	5	1